# A Hybrid Preaching Optimization Algorithm Based on Kapur Entropy for Multilevel Thresholding Color Image Segmentation

**DOI:** 10.3390/e23121599

**Published:** 2021-11-29

**Authors:** Bowen Wu, Liangkuan Zhu, Jun Cao, Jingyu Wang

**Affiliations:** School of Mechanical and Electrical Engineering, Northeast Forestry University, Harbin 150040, China; bwwu@nefu.edu.cn (B.W.); zdhcj@126.com (J.C.); wangjy@nefu.edu.cn (J.W.)

**Keywords:** heuristic algorithm, color image segmentation, Kapur entropy, preaching optimization algorithm, distributed time-delay, evolutionary state

## Abstract

Multilevel thresholding segmentation of color images plays an important role in many fields. The pivotal procedure of this technique is determining the specific threshold of the images. In this paper, a hybrid preaching optimization algorithm (HPOA) for color image segmentation is proposed. Firstly, the evolutionary state strategy is adopted to evaluate the evolutionary factors in each iteration. With the introduction of the evolutionary state, the proposed algorithm has more balanced exploration-exploitation compared with the original POA. Secondly, in order to prevent premature convergence, a randomly occurring time-delay is introduced into HPOA in a distributed manner. The expression of the time-delay is inspired by particle swarm optimization and reflects the history of previous personal optimum and global optimum. To better verify the effectiveness of the proposed method, eight well-known benchmark functions are employed to evaluate HPOA. In the interim, seven state-of-the-art algorithms are utilized to compare with HPOA in the terms of accuracy, convergence, and statistical analysis. On this basis, an excellent multilevel thresholding image segmentation method is proposed in this paper. Finally, to further illustrate the potential, experiments are respectively conducted on three different groups of Berkeley images. The quality of a segmented image is evaluated by an array of metrics including feature similarity index (FSIM), peak signal-to-noise ratio (PSNR), structural similarity index (SSIM), and Kapur entropy values. The experimental results reveal that the proposed method significantly outperforms other algorithms and has remarkable and promising performance for multilevel thresholding color image segmentation.

## 1. Introduction

Image segmentation is a vital processing stage in object location and pattern recognition [1]. It can be deemed as a technique that partitions the components of an image into several disjoint categories concerning color, feature, texture, etc. More precisely, this work can be divided into color image segmentation and gray image segmentation. Color images provide more abundant information than gray images, such as hue and saturation [2]. Hence, color image segmentation has been widely applied in numerous domains such as biological monitoring [3], automatic driving [4], and precision agriculture [5], which makes color image segmentation a demanding task.

In the past few years, researchers have proffered a range of methods to achieve image segmentation, which can be summarized as threshold-based method [6], edge-based method [7], region-based method [8], clustering-based method [9], turbopixel/superpixel-based methods [10,11], watershed-based methods [12,13], contour models-based [14,15], and artificial neural network-based methods [16]. The threshold technique has become the most in vogue method compared with other methods for its simple implementation and high accuracy [17]. It consists of bi-level and multilevel segmentation depending on the number of thresholds. Bi-level segmentation means that the given image should be segmented into two classes concerning a single threshold value, namely target and background [18]. However, the effect of bi-level threshold segmentation is inadequate when the image contains more objects or complex background. Considering these limitations, multilevel segmentation can be adopted. It divides pixels into several regions and can be efficaciously used for color image segmentation.

Numerous techniques based on respective criteria have been developed for getting appropriate thresholds (by way of illustration, Otsu and information entropy). As a primary and available method, Otsu has long been highly valued and applied [19]. In addition, methods based on information entropy are extensively concerned due to captivating mathematical concepts, such as Shannon entropy [20], fuzzy entropy [21], Tsallis entropy [22], Renyi entropy [23], and Kapur entropy [24]. Among them, Kapur entropy classifies an image into multiple classes by comparing the entropy of the histogram. Ergo, Kapur entropy is not sensitive to the size of the subregions and preserves the details better compared with Otsu and other methods. It has been extensively used in multilevel image segmentation [6].

The essence of image segmentation can be regarded as an optimization problem. Still, the computation complexity increases explosively with the number of thresholds increases. For this reason, researchers combine heuristic algorithms with image segmentation methods creatively. In [24], an improved fundamental ant colony optimization with horizontal and vertical crossover search was applied to image segmentation with a non-local means 2D histogram and Kapur entropy. The hybrid method obtained achieved better threshold values with better stability than the original, but its complexity inevitably increased due to the excessive mutation mechanism. In [25], an efficient methodology for multilevel segmentation has been proposed using the Harris Hawks optimization algorithm and the minimum cross-entropy as a fitness function. The experiments conducted in this approach merely considered low-dimensional optimization problems and were only able to handle gray images. In [26], an improved marine predators algorithm has been introduced for COVID-19 images detection with Kapur entropy and outperformed all other algorithms for a range of metrics. The approach outperforms comparative algorithms on high-dimension segmentation, but it suffers from a low convergence rate, which makes it perform poorly in the case of insufficient time. In [27] a crow search algorithm was used for maximizing the Kapur method to tackle the problems of multi-thresholding. The suggested method has fewer parameters to tune and achieved comparatively better results while tested on a set of benchmark images using multiple threshold values. Despite success in this work, it suffers from slow convergence. Additionally, there have been many intelligent optimization algorithms applied to the field of threshold segmentation, such as the sine cosine algorithm [28], sparrow search algorithm [29], particle swarm optimization [30], and multiverse optimization algorithm [31]. To sum up, these studies combine heuristic algorithms with image segmentation methods successfully. Nonetheless, there is still much room for improvement in precision, since the results obtained by approximate optimization algorithms are often not right on the mark enough.

The preaching optimization algorithm (POA) is a novel meta-heuristic algorithm proposed in 2021, which simulates the behavior of preachers in religious communication [32]. Preachers spread successors to improve the search range and select the next generation by the elite mechanism and weight consisted of fitness and location. As reported by experiments in a series of benchmark functions and applications in grayscale images segmentation, POA exhibits better performances than slap swarm algorithm (SSA) [33], grey wolf optimizer (GWO) [34], improved fruit fly optimization algorithm (FFO) [35], dynamic particle swarm optimization algorithm (PSO) [36], firefly algorithm (FL) [37], improved bat algorithm (BA) [38], harris hawks optimization (HHO) [39], moth flame optimization algorithm (MFO) [40], multiverse optimizer (MVO) [41], and whale optimization algorithm (WOA) [42].

As the no free lunch (NFL) theorem for optimization is proposed [43], people realize the openness of the research field of optimization algorithms: there is no ideal algorithm for all problems. More precisely speaking, any heuristic algorithm has its limitations and should solve different domain problems through improvement and adjustment. One of the most remarkable and general-purpose choices is the time-delay strategy. As a physical phenomenon in dynamics, time-delay is of great significance for the algorithm to make full use of its historical information. There have been many algorithms that achieved better execution results by adding time delay (see e.g., [44,45,46]). According to the way in which it occurs, time-delay can be categorized as time-varying, constant, discrete, and distributed in [47]. Among them, distributed time-delay exhibits a distinct spatial nature that models delays in signal propagation distributed through several parallel channels in a certain period. Compared with others, distributed time-delay obtains more historical information and shows more complex dynamic mechanisms, which have been well studied [45,47].

However, as with the above-mentioned heuristic algorithms, there are also some drawbacks of the standard POA algorithm mentioned as follows: unbalanced exploration-exploitation and easy to fall into local optimization. According to the above research of the successful employment of distributed time-delay, a natural idea is to introduce it into POA to enhance the performance to a certain extent. In [48], the mechanism of learning from both individual and global optimal individuals in particle swarm optimization (PSO) is used to express time-delay, which gives us another inspiration to improve POA efficiently, namely hybridization. Simultaneously, how to properly deploy time-delay at each stage is also considerable, and an inventive idea is to implement based on the evolutionary state. The evolutionary state is determined by the evolutionary factor, and the position equations are updated according to it. In the combination with time-delay, this procedure can be understood that the evolutionary state determines which historical information the individual learns more, the global optimal or the individual optimal [49]. Furthermore, for the sake of balanced exploration and exploitation, the distributed time-delay ought to be generated randomly based on a certain probability.

Motivated by the above discussions, the purpose of this paper is to propose an HPOA-based segmentation algorithm for color images. The main contributions of this paper can be summarized as follows:

(1) A novel HPOA algorithm is proposed where (a) the distributed time-delay contributes to a substantial reduction of premature convergence; (b) the hybridizing with PSO provides a thorough exploration of the entire search space; (c) the evolutionary state supplies a significant balance between the local and global search abilities.

(2) An HPOA-based color image segmentation algorithm is obtained by combining HPOA with the Kapur entropy algorithm. The proposed HPOA-based segmentation algorithm searches for a more exact threshold thereby facilitating a better components partition of the color image.

(3) The performances of HPOA and HPOA-based color image segmentation algorithm are investigated in detail: (a) eight classical single-objective benchmark functions are employed to assess the performance of HPOA on various types of problems. (b) A total of 24 Berkeley color images are utilized to verify the effectiveness of the HPOA-based segmentation algorithm on multiple complex images.

The remainder of this article is organized as follows: Section 2 gives an overview of the POA algorithm. Section 3 describes the hybrid algorithm HPOA. Section 4 presents a color image segmentation algorithm based on HPOA. Section 5 introduces the simulation results of HPOA on the benchmark functions, Section 6 illustrates the experiment results of the HPOA-based segmentation method. Section 7 puts forward the conclusion and future work.

## 2. POA Algorithm

POA is a novel swarm intelligence algorithm proposed in 2021 [32]. The main inspiration of the algorithm is the process of religious spread: communication, competition, and development, which will be introduced at length in the following subsections.

### 2.1. Religious Inheritance

When a preacher inherits the religious knowledge to his inheritors, the inheritors will follow around him as follows:

(1) $$loc^{\prime }=loc+randv(r/3)$$

where loc and $loc^{\prime }$ are the positions of preachers and inheritors; randv(r/3) represents a vector following normal distribution with the mean of 1 and variance of r/3; r represents the weighted and normalized fitness value.

### 2.2. Religious Competition

Cultural competition occurs between all the inheritors. The elite individuals with the best fitness are selected directly to the next generation, and the remaining are considered according to the comprehensive ranking of location distribution and fitness function:

(2) $$w=\;distance\times exp(\frac{\;\;\;\;f-\;min\;+q}{max\;-\;min\;+q})$$

where w represents the weight factors to sort individuals; distance is the normalized Euclidean distance from each inheritor to the center of the individual; f represents the fitness, max−min represent the difference between the maximum and minimum fitness values; q is the precision of floating-point numbers.

### 2.3. Religious Development

The new preachers will develop their religion through study tours. The idea of the Levy-flight and normal distribution are employed to implement the behaviors. If they get a better position, then the original position will be updated.

(3) $$loc^{\prime \prime }=\left\{ \begin{array}{l} loc^{\prime }\;+\;0.01\times \;levy\\ loc^{\prime }\;+\;0.01\times \;randv \end{array}\right.$$

where $loc^{\prime \prime }$ is the updated position; randv represents a vector following normal distribution; and the position with higher fitness value will be chosen; levy represents the search step size generated using a levy flight mechanism.

Although the POA algorithm outperforms various popular algorithms in solving engineering problems, there are still some defects existing such as insufficient thorough exploration for the entire search space and easy to fall into local optimum when facing practical problems, thus it needs to be improved.

## 3. A Novel HPOA Algorithm

In this section, a novel HPOA algorithm is proposed to further strengthen the competence of the traditional POA algorithm. The main innovation of this algorithm lies in the introduction of the distributed time delay and the hybridization of the PSO algorithm. In the interim, the evolutionary state strategy is utilized. More specifically, the proposed HPOA algorithm enables the search agents to learn from historical personal or global optimal depending on their evolutionary states. Compared with other algorithms, HPOA pursues stronger capability, avoids being trapped by local optimum, and maintains a balance between convergence and diversity.

### 3.1. Evolutionary State Estimation

The search process of heuristic algorithms is frequently phased. For example, agents are more likely to explore promising areas in the early stage of the search, and more inclined to exploit discovered solutions and potentially surrounding areas in the later stage of optimization. According to [50,51], this kind of behavior should ensure that the algorithm finally converges to the optimal solution in the entire search space. In [49], the evolutionary state of the population is creatively divided into the following four categories: exploration, exploitation, convergence, and escape. It can be represented by State 1–4, respectively, in this paper.

When a biota explores the search space, the distance between different individuals can be used to measure the search state of the population as a whole part. To give an illustration, when the individuals are scattered far away, it means that they are looking for prey. When they gather closer, it’s besieging the excellent targets. The distance $D_{i}$ can be calculated as follows:

(4) $$D_{i}=\frac{1}{Sa-1}\sum_{j=1,j\neq i}^{Sa}\sqrt{{\left(x_{i}-x_{j}\right)}^{2}}$$

where Sa represents the number of populations; $x_{i}$ indicates the position of the individual i.

Then the best individual is selected and its distance $d_{best}$ can be normalized as $D_{n}$:

(5) $$D_{n}=\frac{d_{best}-d_{\min}}{d_{\max}-d_{\min}}$$

where $d_{\max}$ and $d_{\min}$ represent the maximum and minimum values of distance.

Finally, the evolutionary state of the population is classified:

(6) $$State=\left\{ \begin{array}{l} 1,\;\;0.00\leq D_{n}<0.25\\ 2,\;\;0.25\leq D_{n}<0.50\\ 3,\;\;0.50\leq D_{n}<0.75\\ 4,\;\;0.75\leq D_{n}<1.00 \end{array}\right.$$

The estimation of the evolutionary state enables individuals to evaluate their search ability accurately. In the following part, adaptive search strategies will be added for different individuals based on their evolutionary states.

### 3.2. Distributed Time-Delay Based on PSO Idea

In religious competition sessions, inheritors are comprehensively considered to decide whether to become new preachers or not. The new preachers selected by this procedure only consider the fitness and location of an individual in the current iteration. Still and all, the procedure ignores the exchange of information with themselves and the population experience. The search process can be more accurate and efficient if we can make better use of the previous knowledge that individuals and other individuals have searched. As a consequence, the randomly distributed time-delay is introduced into the search model. This strategy enables preachers to enhance the use of the accumulated with better accuracy and pursue a stronger capability of avoiding local trapping problems. Hence, the idea of distributed time-delay based on the PSO algorithm is introduced to the location update formula of POA.

(7) $$loc(t+1)=loc(t)+p_{l}r_{1}\sum_{\tau =1}^{N}\left(loc_{l}(t-\tau )-loc(t)\right)+p_{g}r_{2}\sum_{\tau =1}^{N}\left(loc_{g}(t-\tau )-loc(t)\right)$$

where t represents the number of iteration, $r_{1}$ and $r_{2}$ are random numbers, $p_{l}$ and $p_{g}$ are weighting factors which control the search direction according to the state of evolution, τ is the current delay iterations, and N is the upper bound number of time-delay.

### 3.3. Adaptive Orientation Adjustment Strategy Based on Evolutionary State

On the basis of the evolutionary state and distributed time-delay proposed above, this paper proposes a new location update strategy for POA. The novel orientation adjustment strategy consists of four different states, depending on different directions of historical information. The advantage of the method is to make individuals with adaptive capabilities more targeted to search and keep a proper balance between exploration and exploitation. 

#### 3.3.1. State 1: Exploration

In the exploration state, preachers are expected to search the entire search space as comprehensively as possible to get more optimal solutions. The historic global optimal solution contains a wealth of information, which is distributed in different locations in the search space. Therefore, the distribution time-delay which leads to randomly selected global optimal solutions is added to preachers’ position update formula. Eventually, the orientation adjustment factor is set as $p_{l}=0$ and $p_{g}=0.01$.

#### 3.3.2. State 2: Exploitation

In the exploitation state, preachers should focus on the optimal solution which has been found. They improve search efficiency by learning from the individual optimal solution quickly. The record of the preachers’ behavior in a valuable location is stored in the individual optimal solution, which can improve the search efficiency of the new individual greatly. Therefore, the distribution time-delay which leads to randomly selected individual optimal solutions is added to preachers’ position update formula. The orientation adjustment factor is set as $p_{l}=0.01$ and $p_{g}=0$.

#### 3.3.3. State 3: Convergence

In the convergence state, preachers are encouraged to gather around into the global optimal region as soon as possible. To implement this procedure, they ought to reduce the proximity of the other direction, which can be indicated by setting the orientation adjustment factor in the other direction to zero. The orientation adjustment factor is set as $p_{l}=0$ and $p_{g}=0$.

#### 3.3.4. State 4: Escape

In the escape state, preachers are trying to escape from the region around the local optimal. As a consequence, they learn from the entire history to make enough movement. This procedure expresses by double approximation to the individual and global optimal. The orientation adjustment factor is set as $p_{l}=0.01$ and $p_{g}=0.01$.

**Figure 1:**
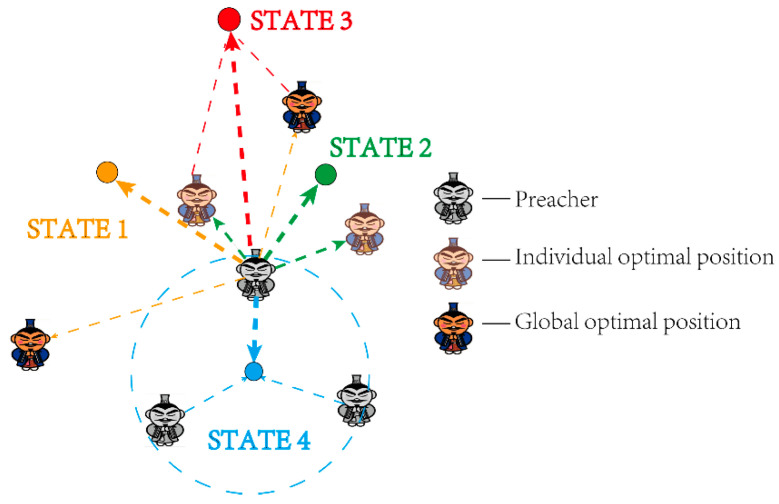
Orientation adjustment strategy of HPOA.

It is worth noting that since the proposed strategy ought to be combined with iteration-based algorithms, it needs historical iteration messages of the individuals as the current agents are guided by previous optimums. The mechanism is shown in Figure 1.

### 3.4. The Framework of the HPOA

The implementation process of HPOA is as follows:

(1) Initialize the parameters including the population size, the max number of iterations, the search dimension, the number of inheritors, and the number of elite individuals.
(2) Initialize the population.
(3) Record global and local optimal historical information.
(4) Transmit the location information to inheritors using Equation (1).
(5) Select new preachers by the mechanism of religion competition using Equation (2).
(6) Estimating the evolutionary state of the new preachers using Equations (4)–(6).
(7) Adding distributed time-delay to the preachers based on their evolutionary state.
(8) Search for a new solution by the mechanism of religion development by Equation (3).
(9) Repeat Steps 3 to 5 till the algorithm meets the max number of iterations.
(10) Output the result.

## 4. HPOA-Based Segmentation Algorithm

In this section, the HPOA algorithm is utilized to optimize the basic Kapur entropy algorithm to improve the defects. Firstly, we present a brief description of the multilevel thresholding segmentation. Then, we describe the Kapur entropy and the fitness function. In the end, we present the proposed HPOA-based color image segmentation method.

### 4.1. Multilevel Thresholding Image Segmentation

Multilevel thresholding segmentation utilizes a threshold group to divide the pixels of each gray level into different categories. This method can not only distinguish the foreground and background of the image but also achieve great results when the image is complex and need to be extracted. In the interim, this method is also suitable for color images. In this method, if the threshold group describe as $\left[t_{1},t_{2},\ldots ,t_{n}\right]$, then the grayscale maps are given as follows:

(8) $$f=\left\{ \begin{array}{l} l_{0},0\leq f\leq t_{1}\\ l_{1},t_{1}\leq f\leq t_{2}\\ \;\vdots \;\;\;\;\;\;\;\;\;\;\;\;\vdots \\ l_{n-1},t_{n-1}\leq f\leq t_{n}\\ l_{n},\;t_{n}\leq f\leq L-1 \end{array}\right.$$

where $l_{0},l_{1},\ldots ,l_{n}$ are the categories of the segmented image; L=256.

### 4.2. Kapur Entropy

Kapur entropy is an automatic threshold selection technique based on the maximization of entropy. It has clear mathematical meaning and can retain the small details excellent, which makes it extensively applied in complex image segmentation. Assuming that n thresholds are selected, then the objective function can be defined as:

(9) $$H(t_{1},t_{2},\ldots ,t_{n})=H_{0}+H_{1}+\ldots +H_{n}$$

where:

(10) $$\begin{array}{l} H_{0}=-\sum_{j=0}^{t_{1}-1}\frac{p_{j}}{\omega_{0}}\ln\frac{p_{j}}{\omega_{0}},\omega_{0}=\sum_{j=0}^{t_{1}-1}p_{j}\\ H_{1}=-\sum_{j=t_{1}}^{t_{2}-1}\frac{p_{j}}{\omega_{1}}\ln\frac{p_{j}}{\omega_{1}},\omega_{1}=\sum_{j=t_{1}}^{t_{2}-1}p_{j}\\ H_{n}=-\sum_{j=t_{n}}^{L-1}\frac{p_{j}}{\omega_{n}}\ln\frac{p_{j}}{\omega_{n}},\omega_{n}=\sum_{j=t_{n}}^{L-1}p_{j} \end{array}$$

where $H_{n}$ denotes different categories entropy, $\omega_{n}$ denotes the probability of each kind of pixel and $P_{j}$ denotes the probability of occurrence of pixels with gray value j. To select the optimal threshold combination, the following formula is used to judge:

(11) $$f_{kapur}=\arg\max\{H(t_{1},t_{2},\ldots ,t_{n})\}$$

The combination maximizing $f_{kapur}$ is the optimal threshold group.

### 4.3. Implementation of the HPOA-Based Segmentation Algorithm

**Figure 2:**
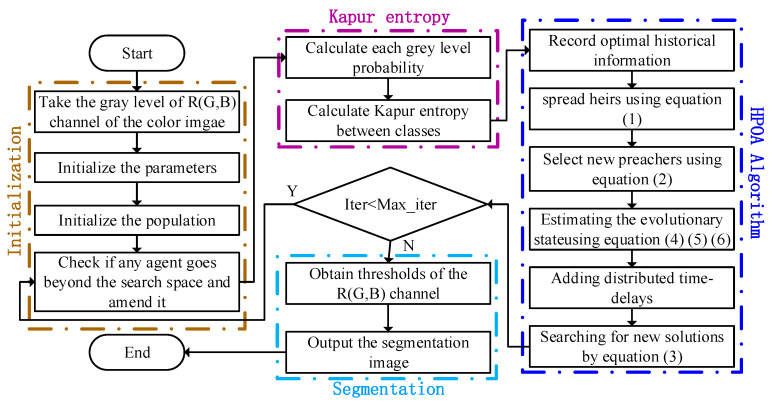
The flow chart of the segmentation algorithm based on HPOA.

To obtain the segmentation threshold more quickly and accurately, the HPOA algorithm is employed to optimize the Kapur entropy. The powerful capability of HPOA obtains more accurate segmentation thresholds, with the segmentation accuracy improving. The flow chart of the proposed segmentation algorithm based on HPOA is shown in Figure 2.

## 5. Simulation and Discussion of the HPOA Algorithm

### 5.1. Selection of Benchmark Functions

To verify the performance of the proposed algorithm, eight well-known benchmark functions are adopted. These functions are categorized into three groups: (1) multimodal, (2) fixed dimension multimodal, and (3) unimodal problems.

**Figure 3:**
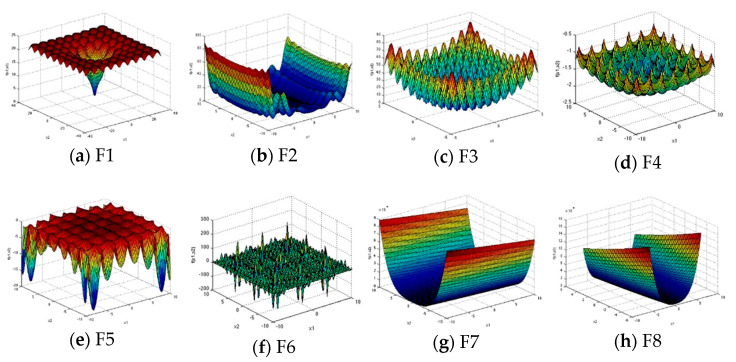
Two−dimensional schematic of benchmark functions.

In the test set, functions F1 to F3 are multimodal with a large number of local optima. The multimodal problems are ordinarily employed to evaluate the exploration ability since a large number of local optima increases the probability of stagnation; Functions F4 to F6 are multimodal but with low dimensions. The fixed dimension multimodal problems have fewer local optima as the dimension is less as compared to the multimodal problems. These problems examine the balance between local and global search abilities; Functions F7 to F8 are unimodal, only one global optimum is present. The unimodal problems evaluate the capability of exploitation. These functions have been used to evaluate algorithms in [34,40,42,52,53]. The details of functions are shown in Table 1, and Figure 3 shows the two-dimensional shapes of the functions, where the edge colors vary according to the heights.

### 5.2. Experimental Setup

All of the algorithms are developed by using Matlab R2016b and implemented on Windows 7 environment on a computer having Intel CPU @2.20 GHz and 12 GB memory. The proposed HPOA is compared with several well-known heuristic algorithms. Each of them contains different characteristics, including:

(1) Traditional POA algorithm [32].
(2) The state-of-the-art WOA algorithm, in which is flexible and requires fewer parameters to be adjusted [42].
(3) The classical representative of swarm intelligence: PSO [30].
(4) A newly proposed algorithm named SCA, containing several adaptive variables to ensure a balance between exploration and development [28].
(5) A novel natural heuristic algorithm: MVO, designed for engineering structure design [41].
(6) MFO, inspired by moth navigation which has advantages in solving unknown space problems [40].
(7) An interesting algorithm, ALO, along with characteristics of few adjusting parameters and high accuracy [53].

More precisely, the population size of all algorithms is set as 20, and the max number of iterations is 1000. Each algorithm runs 10 times to avoid contingency.

### 5.3. Experimental Results of HPOA

As mentioned above, eight benchmark functions are used to evaluate the performance of the proposed HPOA algorithm. In this paper, the comprehensive performance of the algorithm ought to be analyzed by three kinds of criteria: (1) accuracy, (2) convergence, and (3) statistical analysis.

The accuracy criterion of each algorithm is determined by average value and standard deviation. Table 2 and Table 3 present the performance of the HPOA algorithm with different settings of the upper bound of the distributed time-delay N. As seen from the results, the HPOA algorithm obtains the best comprehensive performance when N=200.

The competitive results between HPOA and other algorithms are discussed as follows.

In terms of accuracy, a higher average value signifies better capability. It is found that HPOA is outstanding to comparison algorithms from Table 4 and Table 5. Specifically, the average value of HPOA is normally lowest for each benchmark function, which indicates the superior capability of HPOA. Simultaneously, a lower value of standard deviation indicates better stability. For the functions F2, F3, F7, and F8, the standard deviation performance of HPOA is also most remarkable. Therefore, the experiment results demonstrate that the proposed algorithm has more accuracy and stability than other algorithms.

**Figure 4:**
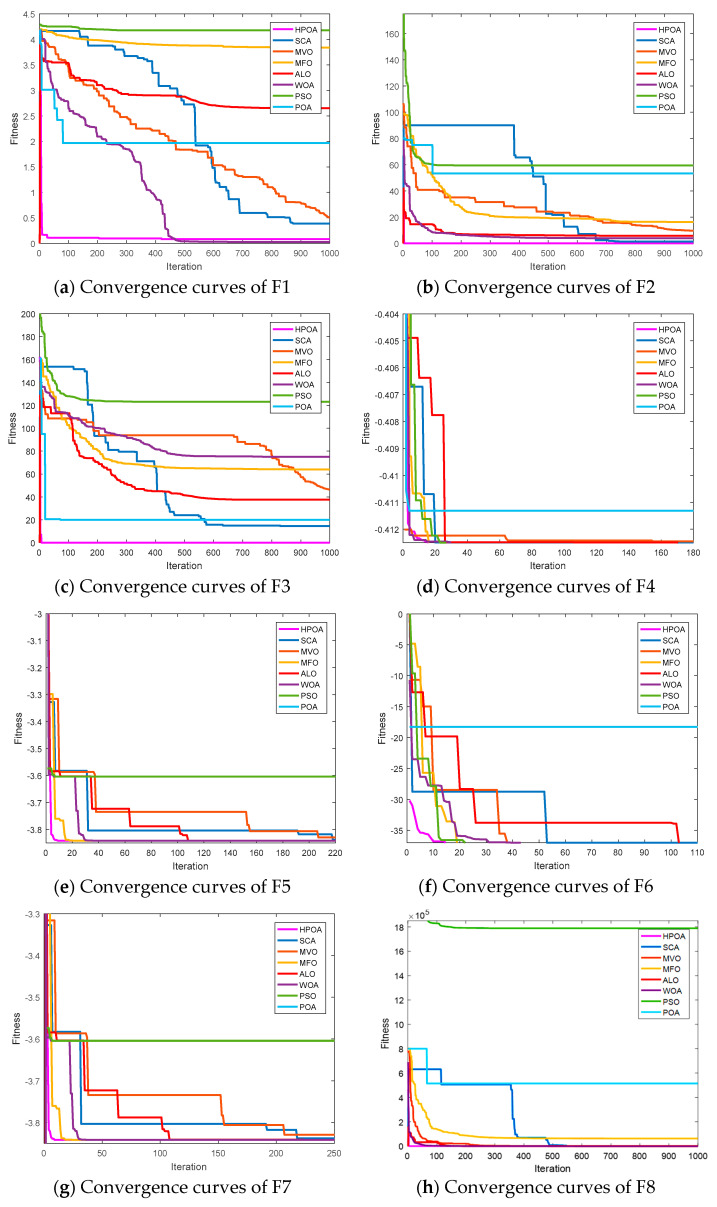
Convergence performance of algorithms.

In terms of convergence, it is observed that HPOA is also the most competitive from Figure 4. For the functions F1, F2, F3, F7, F8, HPOA can converge to the best point most quickly. However, the convergence speed of HPOA is not as fast as POA (for F4 and F6), MVO (for F4), and PSO (for F5). The reason can be found from the fact that the accuracy criterion that this comparison algorithm falls into the local optimal point, which leads to premature convergence. Accordingly, it is demonstrated that the HPOA algorithm has the most extraordinary convergence property.

In terms of statistical analysis, we conduct statistical tests to verify whether the improved algorithm is significantly better than the original algorithm, which is proposed in [50]. And well-established non-parametric tests are applied, namely the Wilcoxon rand sum test. As can be found in Table 6, the proposed new algorithm HPOA has statistical diversity to the comparison algorithm in almost all problems, accounting for 96% of the total. This promising result indicates that HPOA has an astonishing statistical improvement.

Based on above demonstration, HPOA achieves the best performance on N=200, which performs better than seven popular algorithms in various benchmark functions. The experiment results observe that HPOA has better search accuracy, stability, and faster convergence speed. On this basis, there is a significant difference between the proposed algorithm and other methods. Thus, the proposed HPOA algorithm exhibits satisfactory performance, which indicates the reliability of the HPOA-based segmentation algorithm.

## 6. Results and Discussion of the HPOA-Based Segmentation Algorithm

In this section, the HPOA-based segmentation algorithm is employed in color images. The purpose of the experiments is to investigate whether the proposed method is competent in producing high-quality segmented images.

### 6.1. Experimental Setup

We conduct the experiments on three groups of bench images in Table 7 (eight animal images, eight human images, and eight architecture images) from the Berkley Segmentation Dataset and Benchmark 500 (BSDS500). All experiments were performed on the 24 images with the following number of thresholds: 5, 10, 15. This setting enables a more comprehensive comparison of the performance of the proposed algorithm under different dimensions of the problem, which aims to attain more reliable results. Except that the number of iterations is set as 300, the other comparison algorithms and parameter settings are all the same as those in the previous section.

### 6.2. Image Evaluation Metric

The quality of the segmented images can be evaluated by the image evaluation metrics as follows:

#### 6.2.1. Feature Similarity Index (FSIM)

FSIM is an quality assessment (IQA) metrics to measure the image quality automatically [54]. The basic concept for its application in segmentation is evaluating feature similarity between the segmented image and the reference image-the ground truth. FSIM can be calculated as follows:

(12) $$FSIM=\frac{\sum_{x\in X,y\in Y}S_{L}(x,y)\times PC_{m}(x,y)}{\sum_{x\in X,y\in Y}PC_{m}(x,y)}$$

where $S_{L}(x,y)$ is used to evaluate the similarity of the image, $PC_{m}(x,y)$ represents phase congruence of the reference and segmented images, and x∈X,y∈Y represents the pixel domain of an image.

#### 6.2.2. Peak Signal to Noise Ratio (PSNR)

PSNR is a renowned image assessment index, which computes the peak signal-to-noise ratio between two images [55]. This ratio is often used as a quality measurement between the reference and segmented images [56,57], and can be calculated as follows:

(13) $$PSNR=20\log(\frac{255}{RMSE})\;(dB)$$

(14) $$RMSE=\sqrt{\frac{\sum_{i=1}^{H}\sum_{j=1}^{W}\left(I(i,j)-I^{\prime }(i,j)\right)}{H\times W}}$$

#### 6.2.3. Structural Similarity Index (SSIM)

SSIM is an index to measure the similarity of two images, which takes into account various factors such as brightness, contrast, and structural similarity [58]. It finds the similarity between segmented image and the ground truth here:

(15) $$SSIM=l(I,I^{\prime })\times c(I,I^{\prime })\times s(I,I^{\prime })$$

(16) $$c(I,I^{\prime })=\frac{2\sigma_{I}\sigma_{I^{\prime }}+C_{2}}{\sigma_{I}^{2}+\sigma_{I^{\prime }}^{2}+C_{2}}$$

(17) $$S(I,I^{\prime })=\frac{\sigma_{{II}^{\prime }}+C_{3}}{\sigma_{I}\sigma_{I^{\prime }}+C_{3}}$$

(18) $$l(I,I^{\prime })=\frac{2\mu_{I}\mu_{I^{\prime }}+C_{1}}{\mu_{I}^{2}+\mu_{Y}^{2}+C_{1}}$$

where $\mu_{I}$ and $\mu_{I^{\prime }}$ are the mean value of the reference image and the segmented image, $\sigma_{Ι}$ and $\sigma_{{Ι}^{\prime }}$ represents the variance between the images, $\sigma_{Ι{Ι}^{\prime }}$ represent the covariance between the reference image and the segmented image, and $C_{1}$ and $C_{2}$ are constants employed to guarantee the stability.

### 6.3. Experimental Result

To evaluate the result of the algorithms, the quality of the segmented images is quantitatively analyzed by FSIM, PSNR, and SSIM. The proposed method provides segmented results in RGB channels. Considering of these metrics require that the compared images ought to have the same classes, the segmented images are grayed to match the ground truths during the evaluation. The results of FSIM, PSNR, and SSIM of each algorithm are presented in Table 8, Table 9 and Table 10. It can be observed that the proposed algorithm gives excellent results, which are usually the best and the second-best value in three indicators. For instance, in the case of various thresholds for 24 images:

(1) In the FSIM table, the proposed algorithm obtains the most competitive results in almost all cases (66 out of 72 cases). These values indicate the performance of the proposed algorithm is the most outstanding. It is observed that the images segmented by the proposed method have higher similarity and lower distortion with the reference images.

(2) In the PSNR table, although there are only small differences between the algorithms in low dimensions (Dim = 5), HPOA still shows superiority over the others on nearly all the images (21 out of 24 cases). With the number of thresholds increasing, the results become diverse. As exhibited that HPOA can commonly provide the best results as well (Dim = 10, 15), and the PSNR values of HPOA significantly increase.

(3) In the SSIM table, it is perceived that the proposed method outperforms all the other algorithms on various bench images, since the SSIM index obtains the highest values for majority of cases (68 out of 72 cases). The result indicates that the images segmented by IPOA are more similar to the human segmentation images in structural similarity.

Except for the image evaluation indicators, the value of the function fitness can also be a significant index to evaluate the performance of algorithms. Table 11 exhibits the fitness values obtained by each algorithm. It can be perceived that each algorithm can provide a higher fitness value with the increasing of the number of thresholds. The proposed algorithm generally attains better results than the comparison algorithm. The best value and the second-best value achieved by the HPOA-based algorithm are 194 among the 216 problems, accounting for 89% of the total.

**Figure 5:**
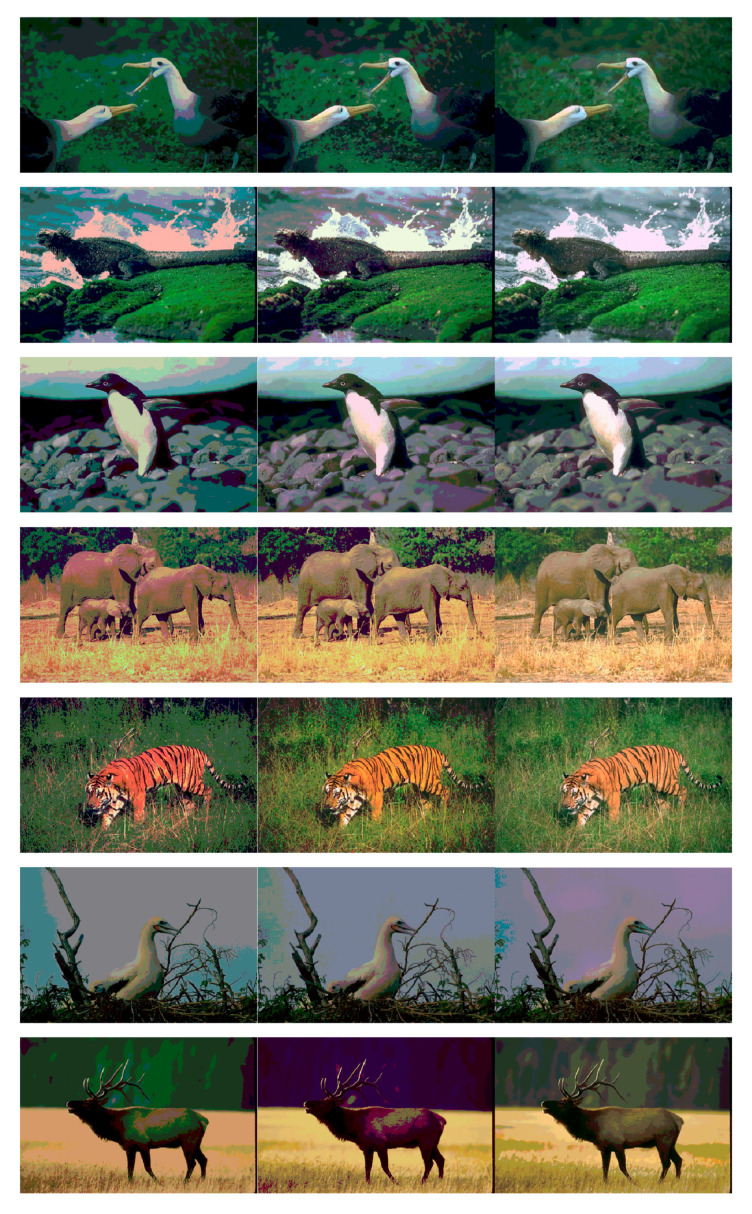

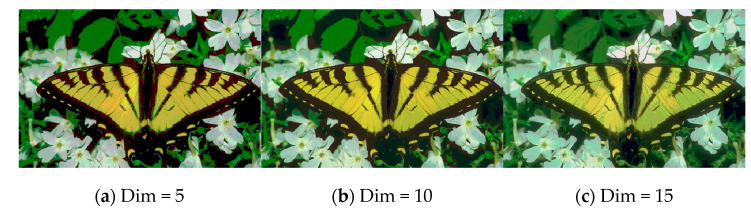
Animal images segmented by proposed method at dim = 5, 10, 15.

**Figure 6:**
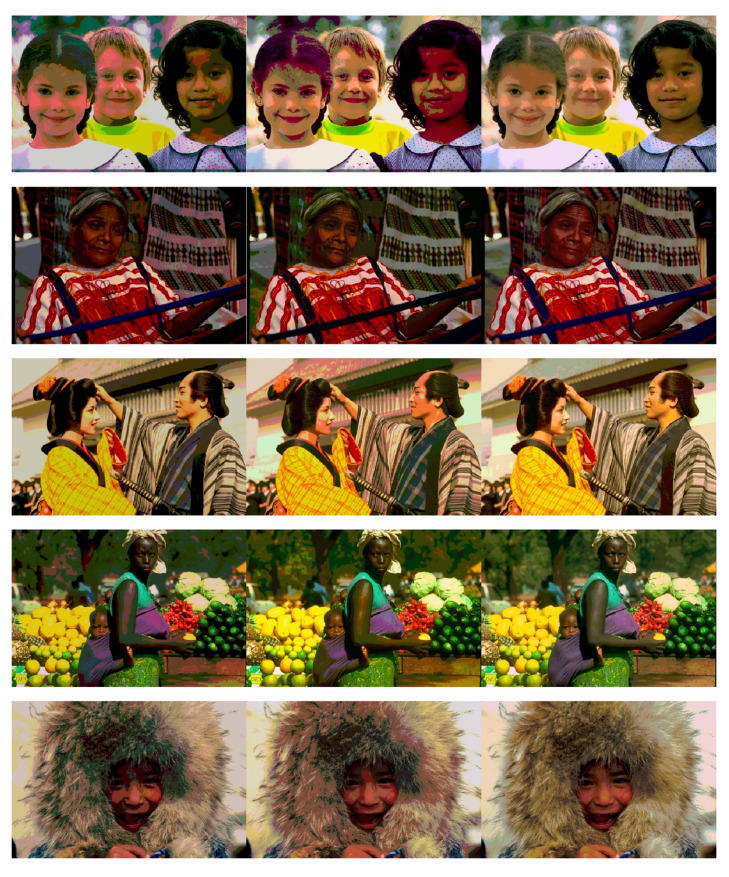

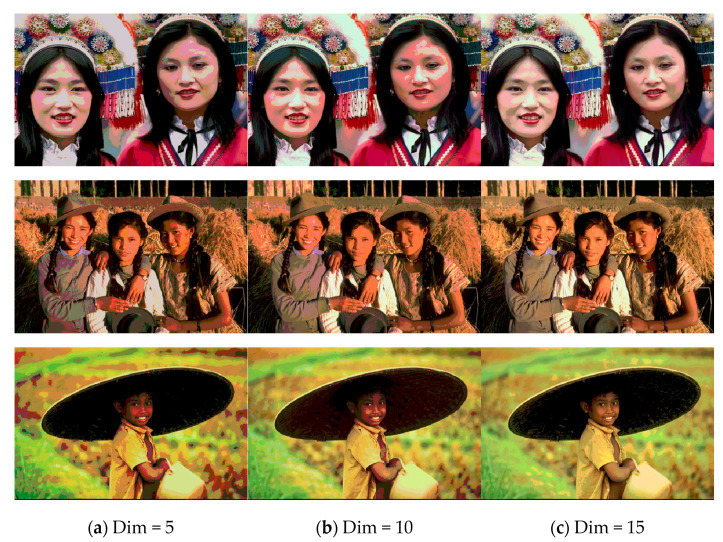
Human images segmented by proposed method at dim = 5, 10, 15.

**Figure 7:**
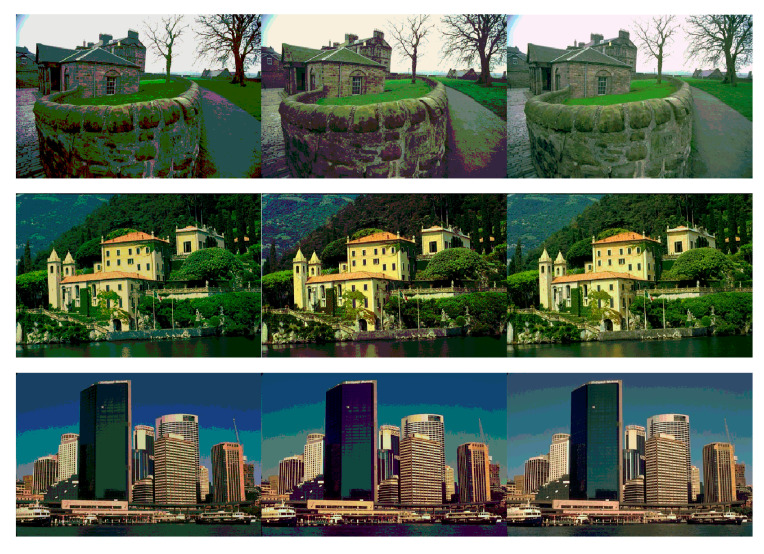

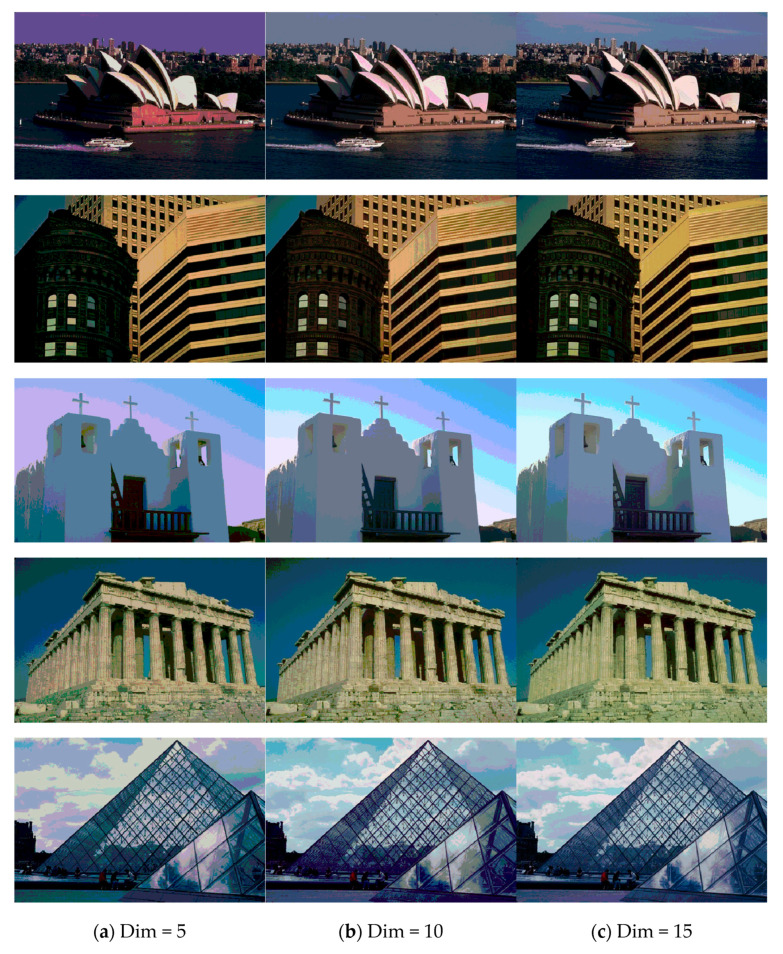
Architecture images segmented by proposed method at dim = 5, 10, 15.

Based on the above demonstration, the competitive values of FSIM, PSNR, SSIM, and fitness values prove the high accuracy of the HPOA-based color image segmentation algorithm. Notably, the superiority of the proposed algorithm becomes more and more remarkable as the number of thresholds increases compared to other algorithms. For this reason, the HPOA-based segmentation algorithm can accomplish the complex tasks of color image segmentation effectively, as well as provide a more precise technique for multilevel segmentation. The segmentation images of the proposed algorithm are shown in Figure 5, Figure 6 and Figure 7.

## 7. Conclusions

This paper presents a hybrid preaching optimization algorithm based on Kapur entropy for complex image segmentation problems. HPOA evaluates the evolutionary state of each population and adjusts the updating model adaptively. Even more noteworthy is that distributed time-delay containing historical information of previous personal and global best is introduced into HPOA. The expression of time-delay draws lessons from the PSO algorithm. Therefore, this strategy strengthens the diversity of the population and prevents premature convergence efficaciously. Eight classical test functions are employed to evaluate the comprehensive performance of HPOA. The validity and stability of the hybrid algorithm are verified by qualitative and quantitative methods. The experiment results reveal that HPOA has better accuracy, stability, and a faster convergence speed than other algorithms. Simultaneously, it has a statistically significant improvement. Eventually, combining HPOA with conventional Kapur entropy, an HPOA-based color image segmentation algorithm is proposed. All segmentation experiments are performed on three categories of images from the Berkeley dataset [59], including eight animal images, eight human images, and eight architecture images. The quality of the segmented images is verified by FSIM, PSNR, SSIM, and Kapur entropy values. These indicators verify that the proposed method also exhibits excellent presentation on various image segmentation problems with strong effectiveness.

As future work, our goal is to further improve HPOA for the MRI image segmentation problem [60]. We also plan to apply the proposed HPOA method for artificial neural network optimization [61] and real-world engineering problems such as structural optimization [62]. Due to the good performance of combing time-delay with the heuristic algorithm, some other efficient methods could be implemented with it for global optimization such as the marine predators algorithm [63] or Harris Hawks optimization algorithm [60]. In addition, considering that the strategy adopted in this paper is an unsupervised processing method, our future work will also focus on the combination with supervised learning mechanisms, such as mask R-CNN [1], including how to improve generalization ability.

## Figures and Tables

**Table 1:** Information of benchmark functions.

Functions	Name	Dimension	Search Space
F1	Ackley Function	d	[−32.768, 32.768]
F2	Levy Function	d	[−10, 10]
F3	Rastrigin Function	d	[−5.12, 5.12]
F4	Cross-in-Tray Function	2	[−10, 10]
F5	Holder Table Function	2	[−10, 10]
F6	Shubert Function	2	[−5.12, 5.12]
F7	Dixon-Price Function	d	[−10, 10]
F8	Rosenbrock Function	d	[−5, 10]

**Table 2:** Mean performance of HPOA with different N.

Functions	HPOA (N = 25)	HPOA (N = 50)	HPOA (N = 75)	HPOA (N = 100)	HPOA (N = 125)	HPOA (N = 150)	HPOA (N = 175)	HPOA (N = 200)
F1	0.52495	0.32073	0.30325	0.36599	0.33331	0.50970	0.39105	0.24924
F2	0.02400	0.02138	0.02263	0.02193	0.02115	0.01104	0.01482	0.01894
F3	0.00022	0.00870	0.00002	0.00000	0.00000	0.00000	0.00000	0.00000
F4	−2.06261	−2.06261	−2.06261	−2.06261	−2.06261	−2.06261	−2.06261	−2.06261
F5	−19.20828	−19.20850	−19.20850	−19.20850	−19.20850	−19.20850	−19.20850	−19.20850
F6	−186.73068	−186.73083	−186.73032	−186.73089	−186.73090	−186.73090	−186.73068	−186.73088
F7	0.40187	0.86632	0.32475	0.66930	0.47461	0.54274	0.39958	0.62513
F8	4.32519	0.00000	0.00000	0.00000	3.45213	4.67038	3.22098	4.85049

**Table 3:** Standard deviation performance of HPOA with different N.

Functions	HPOA (N = 25)	HPOA (N = 50)	HPOA (N = 75)	HPOA (N = 100)	HPOA (N = 125)	HPOA (N = 150)	HPOA (N = 175)	HPOA (N = 200)
F1	0.382	0.223	0.239	0.168	0.234	0.519	0.235	0.277
F2	0.02537	0.02506	0.02657	0.02342	0.02331	0.01878	0.02088	0.02141
F3	0.00055	0.02733	2.51 × 10^−5^	3.68 × 10^−6^	1.62 × 10^−7^	2.84 × 10^−6^	1.27 × 10^−7^	5.94 × 10^−7^
F4	7.95 × 10^−11^	3.49 × 10^−11^	9.26 × 10^−10^	9.82 × 10^−10^	7.64 × 10^−11^	5.05 × 10^−11^	1.31 × 10^−9^	2.43 × 10^−10^
F5	0.00070	4.43 × 10^−8^	3.28 × 10^−7^	4.60 × 10^−7^	4.14 × 10^−7^	7.69 × 10^−7^	1.12 × 10^−6^	6.78 × 10^−7^
F6	0.00036	0.00023	0.00182	1.89 × 10^−5^	1.99 × 10^−5^	1.32 × 10^−5^	0.00056	7.41 × 10^−5^
F7	0.315	0.971	0.237	0.859	0.362	0.379	0.316	0.394
F8	13.6	9.05 × 10^−7^	4.91 × 10^−6^	1.03 × 10^−6^	10.9	14.8	10.2	15.3

**Table 4:** Mean performance of algorithms.

Functions	HPOA (N = 25)	HPOA (N = 50)	HPOA (N = 75)	HPOA (N = 100)	HPOA (N = 125)	HPOA (N = 150)	HPOA (N = 175)	HPOA (N = 200)
F1	0.24924	1.41404	4.16335	18.83677	13.55022	1.51994	20.97066	8.86645
F2	0.01894	19.21614	53.66761	76.44632	21.69743	20.91945	366.39623	288.52300
F3	3.46 × 10^−7^	73.46613	224.93067	377.08160	154.82146	229.48096	624.51340	145.85033
F4	−2.06261	−2.06260	−2.06261	−2.06261	−2.06261	−2.06261	−2.06261	−2.04456
F5	−19.20850	−19.17267	−19.20850	−19.08972	−19.20850	−19.20850	−17.96997	−10.38813
F6	−186.73088	−186.50028	−175.99883	−186.73091	−186.73091	−186.73091	−186.73091	−65.73965
F7	0.625	1.28 × 10^4^	25.3	3.26 × 10^5^	16.7	8.45	6.63 × 10^6^	4.95 × 10^6^
F8	4.85049	2512.80140	80.14614	392,657.86	130.60844	57.3	6.59 × 10^6^	3.33 × 10^6^

**Table 5:** Standard deviation performance of algorithms.

Functions	HPOA (N = 25)	HPOA (N = 50)	HPOA (N = 75)	HPOA (N = 100)	HPOA (N = 125)	HPOA (N = 150)	HPOA (N = 175)	HPOA (N = 200)
F1	0.277	1.42	5.46	1.07	3.49	1.39	0.126	1.65
F2	0.02	14.24	15.49	28.14	7.03	14.08	91.95	15.54
F3	0.00	32.03	31.65	71.59	27.15	101.15	53.60	53.04
F4	2.43 × 10^−10^	1.99 × 10^−5^	6.83 × 10^−9^	4.68 × 10^−16^	6.34 × 10^−15^	3.99 × 10^−15^	4.68 × 10^−16^	0.04
F5	6.78 × 10^−7^	0.03	1.45 × 10^−6^	0.38	4.53 × 10^−13^	8.22 × 10^−14^	1.28	3.77
F6	7.41 × 10^−5^	0.327	33.94	1.64 × 10^−14^	4.88 × 10^−11^	1.24 × 10^−13^	0.00	29.97
F7	0.394	1.39 × 10^4^	23.7	4.58 × 10^5^	10.3	8.50	3.15 × 10^6^	6.72 × 10^5^
F8	15.3	2.43 × 10^3^	50.23	272,823.47	74.38	14.51	1.76 × 10^6^	484,632.28

**Table 6:** Wilcoxon rank comparison of algorithms (h= 1 represents significant difference).

Functions	HPOA vs. SCA		HPOA vs. MVO		HPOA vs. MFO		HPOA vs. ALO		HPOA vs. WOA		HPOA vs. PSO		HPOA vs. POA	
	*p*-Valve	h	*p*-Valve	h	*p*-Valve	h	*p*-Valve	h	*p*-Valve	h	*p*-Valve	h	*p*-Valve	h
F1	0.037635	1	0.000183	1	0.000183	1	0.000183	1	0.011330	1	0.000183	1	0.000183	1
F2	0.000183	1	0.000183	1	0.000183	1	0.000183	1	0.000183	1	0.000183	1	0.000183	1
F3	0.000183	1	0.000183	1	0.000183	1	0.000183	1	0.000183	1	0.000183	1	0.000183	1
F4	0.000183	1	0.000246	1	0.000064	1	0.000504	1	0.000242	1	0.000064	1	0.000183	1
F5	0.000183	1	0.121225	0	0.002036	1	0.000183	1	0.000173	1	0.471171	0	0.000183	1
F6	0.000183	1	0.004586	1	0.000129	1	0.000769	1	0.000173	1	0.000141	1	0.000183	1
F7	0.000183	1	0.000183	1	0.000183	1	0.000183	1	0.000183	1	0.000183	1	0.000183	1

**Table 7:** Original benchmark images and the corresponding histograms.

Original Image	Histogram	Original Image	Histogram
**Animal**			
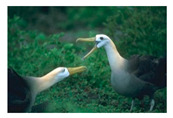	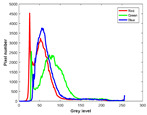	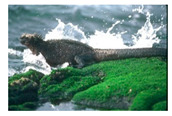	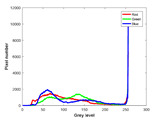
(a) P1-1		(b) P1-2	
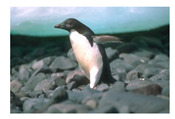	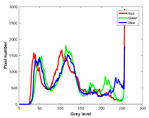	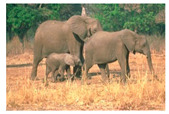	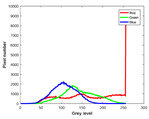
(c) P1-3		(d) P1-4	
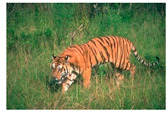	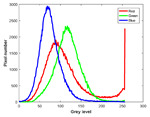	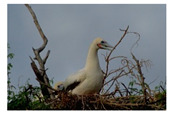	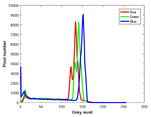
(e) P1-5		(f) P1-6	
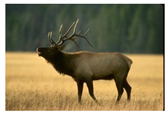	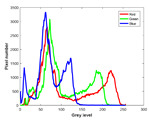	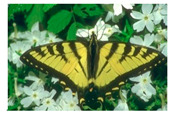	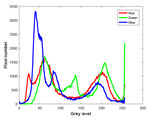
(g) P1-7		(h) P1-8	
**Human**			
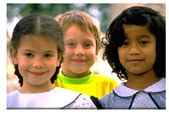	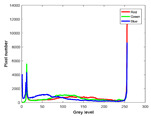	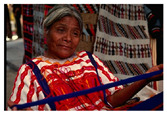	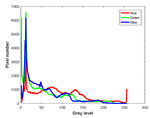
(i) P2-1		(j) P2-2	
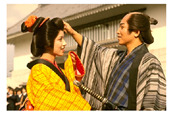	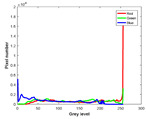	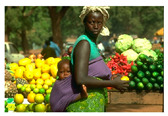	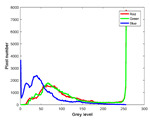
(k) P2-3		(l) P2-4	
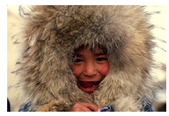	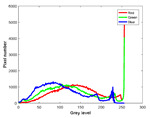	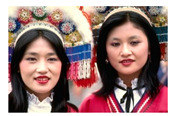	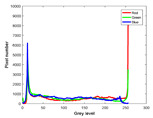
(m) P2-5		(n) P2-6	
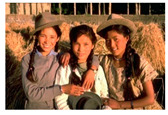	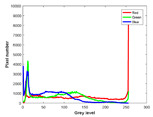	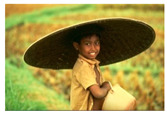	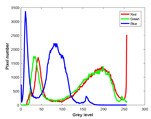
(o) P2-7		(p) P2-8	
**Architecture**			
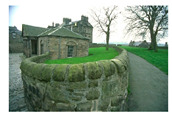	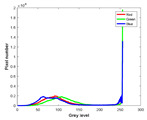	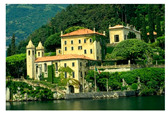	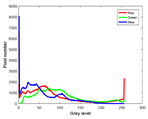
(q) P3-1		(r) P3-2	
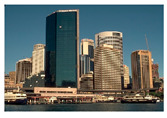	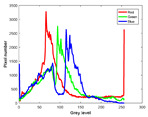	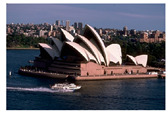	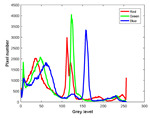
(s) P3-3		(t) P3-4	
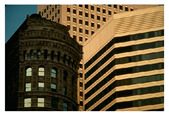	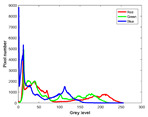	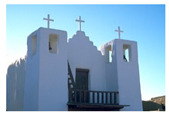	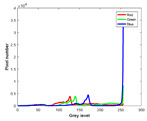
(u) P3-5		(v) P3-6	
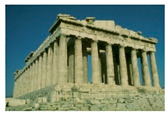	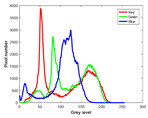	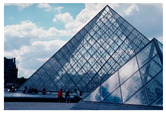	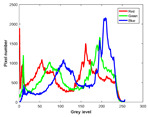
(w) P3-7		(x) P3-8	

**Table 8:** FSIM performance of algorithms.

Image	Dim	HPOA	SCA	MVO	MFO	ALO	WOA	PSO	POA
P1-1	5	0.40	0.37	0.38	0.38	0.38	0.37	0.38	0.35
	10	0.47	0.46	0.43	0.42	0.43	0.43	0.42	0.44
	15	0.43	0.43	0.41	0.42	0.42	0.41	0.42	0.39
P1-2	5	0.50	0.47	0.49	0.50	0.49	0.48	0.48	0.37
	10	0.55	0.52	0.53	0.53	0.52	0.52	0.53	0.50
	15	0.48	0.42	0.46	0.48	0.40	0.42	0.47	0.43
P1-3	5	0.56	0.42	0.54	0.52	0.51	0.51	0.50	0.56
	10	0.58	0.47	0.54	0.54	0.55	0.51	0.56	0.53
	15	0.56	0.48	0.50	0.54	0.55	0.48	0.49	0.51
P1-4	5	0.48	0.47	0.46	0.44	0.46	0.41	0.42	0.42
	10	0.47	0.41	0.43	0.44	0.41	0.46	0.47	0.46
	15	0.53	0.52	0.50	0.52	0.48	0.50	0.50	0.49
P1-5	5	0.48	0.47	0.46	0.46	0.41	0.46	0.42	0.46
	10	0.50	0.47	0.42	0.48	0.41	0.45	0.43	0.40
	15	0.44	0.40	0.42	0.41	0.43	0.41	0.42	0.44
P1-6	5	0.49	0.47	0.43	0.45	0.48	0.48	0.47	0.40
	10	0.55	0.53	0.55	0.53	0.52	0.54	0.54	0.54
	15	0.60	0.60	0.55	0.57	0.57	0.60	0.53	0.54
P1-7	5	0.47	0.41	0.45	0.44	0.44	0.41	0.44	0.42
	10	0.55	0.51	0.52	0.54	0.50	0.52	0.55	0.55
	15	0.61	0.60	0.55	0.55	0.55	0.59	0.60	0.60
P1-8	5	0.45	0.41	0.40	0.41	0.44	0.45	0.42	0.45
	10	0.56	0.54	0.56	0.56	0.56	0.56	0.55	0.50
	15	0.61	0.54	0.60	0.56	0.55	0.59	0.60	0.58
P2-1	5	0.46	0.44	0.46	0.43	0.42	0.41	0.46	0.45
	10	0.58	0.55	0.57	0.51	0.58	0.56	0.51	0.54
	15	0.59	0.56	0.55	0.58	0.55	0.53	0.59	0.55
P2-2	5	0.57	0.42	0.55	0.54	0.51	0.57	0.50	0.50
	10	0.58	0.50	0.53	0.54	0.58	0.56	0.55	0.53
	15	0.61	0.60	0.58	0.61	0.59	0.56	0.57	0.54
P2-3	5	0.54	0.42	0.54	0.52	0.51	0.53	0.52	0.54
	10	0.55	0.52	0.55	0.56	0.56	0.53	0.51	0.52
	15	0.62	0.60	0.56	0.57	0.55	0.54	0.54	0.59
P2-4	5	0.56	0.55	0.49	0.50	0.49	0.50	0.48	0.55
	10	0.58	0.52	0.53	0.51	0.50	0.57	0.51	0.51
	15	0.61	0.57	0.61	0.60	0.59	0.60	0.58	0.60
P2-5	5	0.49	0.43	0.44	0.42	0.40	0.47	0.42	0.42
	10	0.58	0.52	0.51	0.56	0.51	0.57	0.55	0.51
	15	0.63	0.57	0.56	0.61	0.54	0.60	0.55	0.54
P2-6	5	0.46	0.46	0.42	0.45	0.46	0.45	0.45	0.40
	10	0.57	0.58	0.58	0.57	0.56	0.51	0.55	0.53
	15	0.62	0.60	0.55	0.59	0.58	0.61	0.60	0.59
P2-7	5	0.49	0.41	0.47	0.44	0.45	0.44	0.47	0.46
	10	0.59	0.55	0.51	0.58	0.57	0.51	0.53	0.54
	15	0.62	0.59	0.61	0.55	0.60	0.58	0.55	0.55
P2-8	5	0.49	0.46	0.42	0.47	0.43	0.47	0.41	0.43
	10	0.58	0.57	0.58	0.57	0.56	0.51	0.53	0.54
	15	0.61	0.58	0.61	0.54	0.57	0.59	0.60	0.57
P3-1	5	0.49	0.42	0.44	0.42	0.46	0.46	0.42	0.47
	10	0.57	0.51	0.50	0.54	0.50	0.57	0.50	0.52
	15	0.61	0.61	0.58	0.54	0.61	0.57	0.60	0.61
P3-2	5	0.53	0.51	0.52	0.49	0.51	0.52	0.46	0.49
	10	0.59	0.58	0.54	0.57	0.59	0.54	0.53	0.55
	15	0.62	0.62	0.60	0.55	0.57	0.63	0.57	0.61
P3-3	5	0.47	0.44	0.41	0.43	0.45	0.45	0.41	0.46
	10	0.56	0.53	0.55	0.53	0.50	0.55	0.55	0.54
	15	0.60	0.54	0.59	0.57	0.57	0.54	0.54	0.54
P3-4	5	0.48	0.42	0.47	0.42	0.47	0.45	0.45	0.42
	10	0.58	0.56	0.52	0.57	0.57	0.55	0.51	0.51
	15	0.60	0.60	0.60	0.60	0.59	0.55	0.61	0.55
P3-5	5	0.48	0.43	0.40	0.42	0.44	0.41	0.46	0.44
	10	0.55	0.51	0.57	0.53	0.53	0.52	0.56	0.57
	15	0.60	0.55	0.61	0.55	0.59	0.57	0.58	0.56
P3-6	5	0.45	0.44	0.45	0.44	0.41	0.43	0.42	0.41
	10	0.58	0.54	0.56	0.54	0.55	0.54	0.54	0.58
	15	0.62	0.56	0.60	0.54	0.58	0.57	0.58	0.58
P3-7	5	0.46	0.46	0.40	0.46	0.44	0.45	0.42	0.41
	10	0.58	0.52	0.51	0.54	0.50	0.57	0.57	0.50
	15	0.62	0.60	0.56	0.58	0.60	0.56	0.56	0.57
P3-8	5	0.49	0.46	0.47	0.41	0.47	0.42	0.47	0.47
	10	0.55	0.55	0.52	0.55	0.52	0.53	0.55	0.51
	15	0.59	0.54	0.56	0.54	0.56	0.58	0.59	0.57

**Table 9:** PSNR performance of algorithms.

Image	Dim	HPOA	SCA	MVO	MFO	ALO	WOA	PSO	POA
P1-1	5	12.87	12.85	12.31	12.72	12.57	12.12	12.34	12.20
	10	13.86	13.33	13.29	13.36	13.57	13.35	13.49	13.86
	15	14.55	13.81	13.77	14.23	13.92	14.55	14.42	14.26
P1-2	5	15.96	15.97	15.70	15.14	15.53	15.55	15.74	15.60
	10	15.99	15.74	15.15	14.81	15.64	14.92	15.25	15.99
	15	14.91	14.59	13.82	14.56	13.66	13.97	14.91	14.06
P1-3	5	16.05	15.61	16.03	16.06	15.80	14.74	15.90	14.62
	10	15.78	14.87	14.41	13.64	13.60	14.39	15.40	15.76
	15	16.49	15.16	15.36	14.27	15.99	14.92	15.40	16.47
P1-4	5	16.41	15.13	15.90	16.40	14.84	15.80	15.98	15.24
	10	15.59	15.59	14.03	14.96	14.24	15.01	13.74	14.79
	15	11.74	11.49	11.19	10.93	9.70	10.85	11.72	10.76
P1-5	5	18.55	15.32	15.80	15.93	15.37	15.71	16.02	15.90
	10	15.53	15.52	14.09	15.05	15.38	13.73	13.83	15.26
	15	12.18	11.37	10.53	9.28	10.96	11.67	10.37	10.24
P1-6	5	13.84	11.27	11.98	13.83	13.80	12.15	13.50	13.46
	10	17.05	12.07	15.77	14.96	17.05	13.60	16.42	15.54
	15	17.89	15.63	14.93	16.32	17.46	16.99	16.48	15.95
P1-7	5	13.97	12.06	13.97	11.18	12.57	11.39	12.42	11.07
	10	16.43	15.45	16.43	11.56	12.95	12.95	15.46	14.95
	15	17.13	17.13	16.55	16.21	15.87	16.68	16.69	14.23
P1-8	5	13.64	12.21	11.33	12.80	13.11	12.13	12.93	13.66
	10	17.06	12.84	12.38	15.25	17.06	12.41	14.08	16.34
	15	18.62	16.22	14.69	17.45	17.35	16.80	18.40	16.88
P2-1	5	15.58	13.96	14.20	15.58	14.45	15.44	15.27	14.50
	10	17.61	15.46	17.72	18.18	16.65	17.03	16.79	15.64
	15	19.31	17.05	16.59	19.10	16.77	17.33	19.30	16.78
P2-2	5	16.46	14.61	15.59	16.58	16.36	16.12	16.49	15.73
	10	20.37	17.26	18.94	17.79	18.45	18.49	20.37	17.29
	15	21.55	17.73	18.86	18.72	21.54	17.35	18.93	17.35
P2-3	5	14.88	13.59	14.28	13.14	14.88	14.65	14.29	13.44
	10	16.57	15.23	16.13	14.92	17.58	16.60	16.03	15.15
	15	20.57	18.72	15.88	15.92	20.56	17.13	16.31	15.92
P2-4	5	16.10	14.69	16.09	15.88	15.72	15.33	14.38	15.28
	10	18.48	16.40	18.21	18.46	17.28	16.83	18.46	16.85
	15	23.55	21.96	23.55	22.97	21.30	22.09	21.04	21.41
P2-5	5	16.42	15.56	14.58	15.72	15.54	16.40	16.36	14.46
	10	19.32	17.10	15.40	18.52	17.26	19.30	17.70	15.83
	15	18.92	16.66	16.01	18.39	17.71	18.92	17.49	16.07
P3-1	5	15.52	15.39	15.51	15.37	14.56	14.02	14.81	13.66
	10	19.54	19.54	17.88	18.55	15.58	16.60	16.31	15.79
	15	22.03	21.89	22.03	21.26	21.48	20.63	21.40	20.72
P3-2	5	11.38	10.73	10.34	9.48	10.28	11.38	9.78	10.03
	10	15.80	15.38	15.16	14.60	15.23	15.80	14.40	14.76
	15	17.88	17.58	17.81	16.25	16.40	17.86	16.14	16.22
P3-3	5	18.55	14.32	14.57	16.40	15.52	16.23	15.74	14.37
	10	19.85	17.01	17.97	19.85	18.97	17.52	19.73	17.24
	15	22.95	21.14	20.74	22.79	22.59	22.42	22.95	20.95
P3-4	5	15.47	13.67	14.62	15.03	15.47	13.48	14.88	14.39
	10	19.13	15.84	18.31	16.94	19.13	15.37	18.22	15.41
	15	21.85	20.77	20.71	21.39	21.85	20.59	21.28	21.05
P3-5	5	12.18	11.06	10.10	10.04	11.46	9.71	9.72	9.50
	10	16.56	16.56	14.27	14.80	15.53	14.09	13.85	14.29
	15	18.17	18.03	16.18	17.70	18.17	16.66	16.48	16.31
P3-6	5	13.68	11.98	12.65	13.81	13.44	11.27	11.39	11.32
	10	17.55	12.56	15.58	17.55	17.11	11.57	12.73	13.46
	15	18.66	17.30	17.40	18.24	16.79	15.52	16.65	16.46
P3-7	5	12.91	12.61	12.98	11.93	11.30	11.53	12.76	11.99
	10	16.45	13.16	14.00	13.11	14.78	13.78	16.04	12.11
	15	18.00	17.43	15.54	14.49	17.06	16.13	17.16	14.14
P3-8	5	13.63	11.92	13.56	11.15	13.63	12.01	12.92	11.66
	10	16.63	15.74	14.95	11.89	16.53	14.72	13.64	13.41
	15	19.48	16.46	19.03	13.49	17.18	15.65	17.78	15.82

**Table 10:** SSIM performance of algorithms.

Image	Dim	HPOA	SCA	MVO	MFO	ALO	WOA	PSO	POA
P1-1	5	0.39	0.30	0.33	0.26	0.34	0.29	0.33	0.33
	10	0.37	0.30	0.34	0.28	0.30	0.29	0.34	0.28
	15	0.35	0.31	0.32	0.26	0.27	0.25	0.28	0.35
P1-2	5	0.33	0.35	0.29	0.31	0.34	0.26	0.27	0.29
	10	0.35	0.33	0.33	0.26	0.34	0.32	0.35	0.29
	15	0.39	0.35	0.30	0.33	0.28	0.31	0.31	0.35
P1-3	5	0.35	0.28	0.27	0.32	0.35	0.34	0.30	0.25
	10	0.38	0.35	0.26	0.27	0.25	0.33	0.25	0.32
	15	0.35	0.33	0.26	0.33	0.28	0.29	0.27	0.26
P1-4	5	0.36	0.33	0.25	0.26	0.30	0.27	0.32	0.31
	10	0.38	0.32	0.31	0.25	0.29	0.35	0.33	0.33
	15	0.32	0.32	0.30	0.27	0.32	0.28	0.26	0.26
P1-5	5	0.38	0.26	0.34	0.27	0.33	0.33	0.32	0.28
	10	0.38	0.27	0.30	0.31	0.29	0.28	0.34	0.31
	15	0.38	0.27	0.26	0.26	0.31	0.33	0.27	0.34
P1-6	5	0.47	0.47	0.36	0.40	0.34	0.45	0.45	0.41
	10	0.56	0.51	0.51	0.56	0.53	0.56	0.47	0.54
	15	0.58	0.58	0.52	0.56	0.54	0.49	0.55	0.54
P1-7	5	0.48	0.44	0.47	0.45	0.38	0.38	0.44	0.42
	10	0.59	0.44	0.43	0.56	0.46	0.54	0.59	0.52
	15	0.57	0.51	0.55	0.56	0.52	0.54	0.46	0.50
P1-8	5	0.43	0.43	0.41	0.41	0.42	0.40	0.40	0.34
	10	0.54	0.53	0.46	0.54	0.47	0.54	0.53	0.50
	15	0.60	0.51	0.54	0.58	0.51	0.48	0.47	0.53
P2-1	5	0.40	0.34	0.29	0.29	0.32	0.29	0.35	0.28
	10	0.44	0.36	0.41	0.38	0.44	0.44	0.35	0.42
	15	0.52	0.41	0.47	0.46	0.49	0.47	0.48	0.48
P2-2	5	0.34	0.34	0.29	0.26	0.27	0.27	0.31	0.28
	10	0.45	0.45	0.44	0.41	0.36	0.40	0.36	0.37
	15	0.50	0.50	0.44	0.48	0.40	0.49	0.42	0.48
P2-3	5	0.38	0.31	0.28	0.30	0.34	0.31	0.30	0.26
	10	0.39	0.44	0.37	0.42	0.39	0.38	0.42	0.44
	15	0.48	0.41	0.43	0.46	0.40	0.41	0.46	0.43
P2-4	5	0.38	0.29	0.28	0.26	0.33	0.26	0.30	0.27
	10	0.45	0.35	0.44	0.45	0.36	0.36	0.42	0.42
	15	0.53	0.47	0.41	0.50	0.48	0.47	0.47	0.47
P2-5	5	0.36	0.28	0.29	0.34	0.28	0.31	0.28	0.34
	10	0.48	0.43	0.43	0.37	0.44	0.41	0.36	0.39
	15	0.53	0.49	0.44	0.49	0.48	0.41	0.47	0.46
P2-6	5	0.51	0.46	0.36	0.38	0.46	0.36	0.49	0.42
	10	0.54	0.49	0.51	0.56	0.47	0.51	0.48	0.47
	15	0.60	0.52	0.54	0.57	0.59	0.51	0.47	0.55
P2-7	5	0.46	0.42	0.38	0.34	0.41	0.45	0.39	0.41
	10	0.57	0.53	0.55	0.49	0.53	0.50	0.50	0.48
	15	0.61	0.54	0.60	0.56	0.49	0.49	0.50	0.53
P2-8	5	0.48	0.40	0.42	0.37	0.43	0.44	0.46	0.35
	10	0.56	0.49	0.53	0.55	0.46	0.53	0.49	0.52
	15	0.61	0.59	0.55	0.53	0.48	0.54	0.48	0.48
P3-1	5	0.38	0.28	0.34	0.26	0.27	0.35	0.32	0.28
	10	0.46	0.45	0.39	0.36	0.42	0.38	0.36	0.45
	15	0.50	0.50	0.50	0.49	0.42	0.42	0.46	0.49
P3-2	5	0.37	0.29	0.28	0.34	0.29	0.32	0.34	0.31
	10	0.45	0.37	0.43	0.38	0.39	0.45	0.44	0.40
	15	0.47	0.47	0.44	0.47	0.43	0.41	0.45	0.42
P3-3	5	0.38	0.28	0.30	0.27	0.34	0.31	0.34	0.27
	10	0.48	0.45	0.41	0.43	0.41	0.41	0.42	0.45
	15	0.50	0.42	0.46	0.48	0.42	0.48	0.50	0.49
P3-4	5	0.37	0.26	0.33	0.29	0.28	0.34	0.28	0.30
	10	0.43	0.43	0.38	0.38	0.45	0.39	0.35	0.43
	15	0.47	0.46	0.48	0.45	0.41	0.45	0.46	0.49
P3-5	5	0.35	0.31	0.34	0.34	0.35	0.25	0.31	0.25
	10	0.43	0.37	0.42	0.41	0.36	0.37	0.40	0.43
	15	0.51	0.46	0.48	0.42	0.49	0.46	0.43	0.41
P3-6	5	0.46	0.32	0.34	0.47	0.45	0.37	0.43	0.41
	10	0.56	0.56	0.46	0.46	0.52	0.46	0.55	0.50
	15	0.57	0.48	0.54	0.56	0.52	0.54	0.49	0.52
P3-7	5	0.47	0.43	0.45	0.47	0.45	0.47	0.36	0.40
	10	0.57	0.54	0.52	0.57	0.45	0.57	0.57	0.48
	15	0.60	0.59	0.58	0.58	0.53	0.47	0.54	0.50
P3-8	5	0.52	0.34	0.50	0.46	0.40	0.44	0.45	0.44
	10	0.56	0.51	0.56	0.50	0.50	0.51	0.54	0.45
	15	0.63	0.53	0.56	0.49	0.61	0.63	0.60	0.57

**Table 11:** Fitness performance of algorithms.

Image	Dim	Channel	HPOA	SCA	MVO	MFO	ALO	WOA	PSO	POA
P1-1	5	R	21.54	21.47	21.54	21.54	21.54	21.53	21.54	20.54
		G	21.44	21.10	21.38	21.38	21.38	21.38	21.38	21.28
		B	21.27	21.04	21.27	21.27	21.27	21.27	21.27	20.57
	10	R	33.29	32.33	33.34	33.35	33.35	32.58	33.35	31.17
		G	32.93	31.33	32.98	32.99	32.98	32.97	32.75	29.94
		B	32.92	31.87	32.89	32.91	32.92	32.66	32.41	31.02
	15	R	43.11	40.68	43.11	42.46	43.10	42.17	42.31	40.11
		G	42.81	39.63	42.54	41.57	42.49	41.77	42.27	38.44
		B	42.47	39.75	42.15	42.13	42.38	42.02	42.27	37.66
P1-3	5	R	21.36	21.13	21.36	21.36	21.36	21.35	21.36	19.64
		G	21.40	21.38	21.40	21.40	21.40	21.39	21.40	21.21
		B	21.68	21.53	21.68	21.68	21.68	21.67	21.68	20.51
	10	R	32.94	31.48	32.89	32.84	32.93	32.63	32.89	30.52
		G	32.88	31.28	32.80	32.85	32.92	32.79	32.93	30.00
		B	33.39	32.67	33.39	33.38	33.37	33.34	33.00	31.36
	15	R	42.47	40.00	42.41	42.41	42.47	42.07	42.06	37.40
		G	42.35	40.13	41.88	42.19	40.62	42.35	41.54	38.12
P1-6	5	R	20.16	19.65	20.08	19.91	20.08	19.90	19.92	17.69
		G	19.75	19.48	19.71	19.71	19.71	19.50	19.60	17.99
		B	19.54	19.35	19.26	19.48	19.48	19.47	19.48	18.65
	10	R	31.10	29.18	30.78	31.09	31.10	30.15	30.65	25.70
		G	30.71	27.96	30.70	30.53	30.65	29.53	30.71	26.61
		B	30.04	28.16	29.95	30.04	29.58	29.59	29.87	24.43
	15	R	39.88	36.47	38.61	38.63	39.83	39.17	39.16	33.31
		G	39.50	36.82	39.48	39.16	39.21	38.45	38.51	32.47
		B	38.31	33.55	38.02	36.84	38.22	35.98	36.34	28.17
P2-2	5	R	22.15	22.03	22.17	22.17	22.17	22.17	22.17	21.18
		G	22.78	21.56	21.83	21.83	21.83	21.83	21.72	21.60
		B	21.57	21.31	21.49	21.49	21.49	21.46	21.06	20.85
	10	R	34.74	33.10	34.32	34.18	34.31	34.25	33.94	31.44
		G	34.23	33.01	34.19	34.23	34.03	34.16	34.03	30.87
		B	34.01	32.52	33.21	33.24	33.29	33.15	33.21	32.71
	15	R	44.29	42.25	44.17	44.14	44.06	43.70	44.18	41.18
		G	44.74	41.83	43.94	43.87	43.83	43.43	43.85	41.12
		B	43.68	40.01	42.57	42.88	42.92	42.84	42.58	39.12
P2-4	5	R	22.07	21.19	21.23	21.23	21.23	21.23	21.23	20.63
		G	21.55	20.97	21.16	21.16	21.16	21.15	21.16	20.58
		B	22.36	22.11	22.20	22.20	22.20	22.20	22.20	20.98
	10	R	33.94	32.92	33.40	33.42	33.42	33.34	33.28	29.80
		G	33.45	32.61	33.14	33.19	33.11	33.20	33.17	30.12
		B	34.68	33.62	34.54	34.54	34.53	34.52	34.53	33.13
	15	R	43.80	41.08	43.38	43.53	43.36	43.08	43.31	40.55
		G	43.65	41.45	43.47	42.85	43.27	42.54	43.15	40.85
		B	44.94	42.41	44.48	44.45	44.54	44.29	44.44	40.14
P2-6	5	R	21.89	21.63	21.80	21.80	21.80	21.80	21.80	21.27
		G	22.05	21.95	22.01	22.01	22.01	22.00	22.01	21.42
		B	22.10	21.92	22.05	22.05	22.05	22.05	22.05	20.77
	10	R	33.78	33.34	33.80	33.80	33.82	32.92	33.66	31.82
		G	34.08	32.41	34.03	34.16	34.17	33.71	34.05	32.90
		B	34.19	33.26	34.08	34.11	34.10	34.15	34.07	32.28
	15	R	43.82	41.03	43.67	43.31	43.78	43.53	43.72	40.81
		G	44.12	41.98	43.80	43.73	44.11	44.01	43.19	41.35
		B	44.20	41.50	44.18	44.14	43.90	43.20	43.86	40.22
P3-1	5	R	20.06	19.75	19.81	19.81	19.81	19.81	19.81	19.11
		G	19.92	19.42	19.50	19.50	19.50	19.50	19.50	18.85
		B	20.60	20.41	20.51	20.51	20.51	20.51	20.48	19.53
	10	R	32.81	31.33	32.15	32.10	32.15	32.05	31.61	29.85
		G	32.03	30.23	31.44	31.52	31.62	31.58	31.62	28.03
		B	33.78	31.06	32.87	32.84	32.84	32.78	32.91	30.86
	15	R	41.91	38.44	41.49	41.75	41.57	41.81	41.91	38.95
		G	41.89	38.60	41.14	41.24	41.41	39.05	41.23	37.60
		B	43.06	40.22	42.95	42.81	43.06	42.15	43.02	40.57
P3-8	5	R	22.23	22.02	22.15	22.15	22.15	22.15	22.15	21.39
		G	22.09	21.97	22.02	22.02	22.02	22.02	22.02	21.35
		B	21.91	21.82	21.91	21.78	21.91	21.89	21.91	21.50
	10	R	34.32	33.77	34.24	34.24	34.32	34.28	34.32	32.51
		G	34.33	33.09	34.29	34.33	34.10	34.10	34.20	32.68
		B	34.30	32.79	34.30	34.22	33.94	33.91	34.13	31.55
	15	R	44.42	41.82	44.34	44.11	44.26	43.98	44.25	41.72
		G	44.61	42.50	44.21	44.51	44.43	44.26	43.08	40.41
		B	44.41	42.32	44.22	44.21	44.41	43.44	43.54	39.91

## Data Availability

The data used to support the findings of this study are available from the first author upon request.

## References

[1] He K., Gkioxari G., Dollar P., Girshick R. (2020). Mask R-CNN. IEEE Trans. Pattern Anal. Mach. Intell..

[2] Bhandari A.K. (2020). A novel beta differential evolution algorithm-based fast multilevel thresholding for color image segmentation. Neural Comput. Appl..

[3] Li K., Qi X., Luo Y., Yao Z., Sun M. (2021). Accurate retinal vessel segmentation in color fundus images via fully attention-based networks. IEEE J. Biomed. Health.

[4] Farhat W., Sghaier H., Faiedh H., Souani C. (2019). Design of efficient embedded system for road sign recognition. J. Ambient Intell. Humaniz. Comput..

[5] Gao G., Xiao K., Jia Y. (2020). A spraying path planning algorithm based on colour-depth fusion segmentation in peach orchards. Comput. Electron. Agric..

[6] Zhao D., Liu L., Yu F., Heidari A.A., Wang M., Oliva D., Muhammad K., Chen H. (2020). Ant colony optimization with horizontal and vertical crossover search: Fundamental visions for multi-threshold image segmentation. Expert Syst. Appl..

[7] He C., Li S., Xiong D., Fang P., Liao M. (2020). Remote sensing image semantic segmentation based on edge information guidance. Remote Sens..

[8] Shao Z., Zhou W., Deng X., Zhang M., Cheng Q. (2020). Multilabel Remote Sensing Image Retrieval Based on Fully Convolutional Network. IEEE J. Sel. Top. Appl. Earth Observ. Remote Sens..

[9] Keuper M., Tang S., Andres B., Brox T., Schiele B. (2020). Motion Segmentation & Multiple Object Tracking by Correlation Co-Clustering. IEEE T. Pattern Anal..

[10] Levinshtein A., Stere A., Kutulakos K.N., Fleet D.J., Dickinson S.J., Siddiqi K. (2009). Turbopixels: Fast superpixels using geometric flows. IEEE Trans. Pattern Anal. Mach. Intell..

[11] Stutz D., Hermans A., Leibe B. (2018). Superpixels: An evaluation of the state-of-the-art. Comput. Vis. Image Underst..

[12] Ciecholewski M. (2015). Automated coronal hole segmentation from Solar EUV Images using the watershed transform. J. Vis. Commun. Image Represent..

[13] Cousty J., Bertrand G., Najman L., Couprie M. (2010). Watershed cuts: Thinnings, shortest path forests, and topological watersheds. IEEE Trans. Pattern Anal. Mach. Intell..

[14] Zhao L., Gao X., Yuan Y., Tao D. (2014). Geometric active curve for selective entropy optimization. Neurocomputing.

[15] Ding K., Xiao L., Weng G. (2017). Active contours driven by region-scalable fitting and optimized Laplacian of Gaussian energy for image segmentation. Signal Process..

[16] Zhou Z., Siddiquee M.M.R., Tajbakhsh N., Liang J. (2020). UNet plus plus: Redesigning skip connections to exploit multiscale features in image segmentation. IEEE T. Med. Imaging.

[17] Breve F. (2019). Interactive image segmentation using label propagation through complex networks. Expert Syst. Appl..

[18] Lang C., Jia H. (2019). Kapur’s Entropy for Color Image Segmentation Based on a Hybrid Whale Optimization Algorithm. Entropy.

[19] Bhandari A.K., Singh A., Kumar I.V. (2021). Spatial Context Energy Curve-Based Multilevel 3-D Otsu Algorithm for Image Segmentation. IEEE Trans. Syst. Man Cybern.-Syst..

[20] Back A.D., Angus D., Wiles J. (2020). Transitive entropy-a rank ordered approach for natural sequences. IEEE J. Sel. Top. Signal Process..

[21] Wu C., Cao Z. (2021). Entropy-like divergence based kernel fuzzy clustering for robust image segmentation. Expert Syst. Appl..

[22] Rahaman J., Sing M. (2021). An efficient multilevel thresholding based satellite image segmentation approach using a new adaptive cuckoo search algorithm. Expert Syst. Appl..

[23] Jalab H.A., Al-Shamasneh A.R., Shaiba H., Ibrahim R.W., Baleanu D. (2021). Fractional renyi entropy image enhancement for deep segmentation of kidney mri. CMC-Comput. Mater. Con..

[24] Zhao D., Liu L., Yu F., Heidari A.A., Chen H. (2021). Chaotic random spare ant colony optimization for multi-threshold image segmentation of 2D Kapur entropy. Knowl.-Based Syst..

[25] Rodriguez-Esparza E., Zanella-Calzada L.A., Oliva D., Heidari A.A., Foong L.K. (2020). An efficient Harris hawks-inspired image segmentation method. Expert Syst. Appl..

[26] Abdel-Basset M., Mohamed R., Elhoseny M., Chakrabortty R.K., Ryan M. (2020). A hybrid covid-19 detection model using an improved marine predators algorithm and a ranking-based diversity reduction strategy. IEEE Access.

[27] Upadhyay P., Chhabra J.K. (2019). Kapur’s entropy based optimal multilevel image segmentation using crow search algorithm. Appl. Soft. Comput..

[28] Gupta S., Deep K. (2019). Improved sine cosine algorithm with crossover scheme for global optimization. Knowl.-Based Syst..

[29] Xue J., Shen B. (2020). A novel swarm intelligence optimization approach: Sparrow search algorithm. Syst. Sci. Control Eng..

[30] Martino F.D., Sessa S. (2020). PSO image thresholding on images compressed via fuzzy transforms. Inform. Sciences.

[31] Jia H., Peng X., Song W., Lang C., Xing Z., Sun K. (2019). Multiverse optimization algorithm based on levy flight improvement for multithreshold color image segmentation. IEEE Access.

[32] Wei D., Wang Z., Si L., Tan C. (2021). Preaching-inspired swarm intelligence algorithm and its applications. Knowl.-Based Syst..

[33] Mirjalili S., Gandomi A.H., Mirjalili S.Z., Saremi S., Faris H., Mirjalili S.M. (2017). Salp swarm algorithm: A bio-inspired optimizer for engineering design problems. Adv. Eng. Softw..

[34] Mirjalili S.M., Mirjalili S., Lewis A. (2014). Grey wolf optimizer. Adv. Eng. Softw..

[35] Baniani E.A., Chalechale A. (2013). Hybrid pso and genetic algorithm for multilevel maximum entropy criterion threshold selection. Int. J. Hydrog. Energy.

[36] Liu Z., Wei H., Zhong Q., Liu K., Xiao X., Wu L. (2017). Parameter estimation for VSI-Fed PMSM based on a dynamic PSO with learning strategies. IEEE T. Power Electr..

[37] Yang X. Firefly algorithms for multimodal optimization. Proceedings of the 5th International Conference on Stochastic Algorithms: Foundations and Applications.

[38] Xu J., Wang Z., Tan C., Si L., Liu X. (2018). Cutting pattern identification for coal mining shearer through a swarm intelligence-based variable translation wavelet neural network. Sensors.

[39] Heidari A.A., Mirjalili S., Faris H., Aljarah I., Mafarja M., Chen H. (2019). Harris hawks optimization: Algorithm and applications. Futur. Gener. Comp. Syst..

[40] Mirjalili S. (2015). Moth-flame optimization algorithm: A novel nature-inspired heuristic paradigm. Knowl.-Based Syst..

[41] Mirjalili S., Mirjalili S.M., Hatamlou A. (2016). Multi-verse optimizer: A nature-inspired algorithm for global optimization. Neural Comput. Appl..

[42] Mirjalili S., Lewis A. (2016). The whale optimization algorithm. Adv. Eng. Softw..

[43] Wolpert D.H., Macready W.G. (1997). No free lunch theorems for optimization. IEEE Trans. Evol. Comput..

[44] Song B., Wang Z., Zou L. (2017). On global smooth path planning for mobile robots using a novel multimodal delayed PSO algorithm. Cogn. Comput..

[45] Tang Y., Wang Z., Fang J. (2011). Parameters identification of unknown delayed genetic regulatory networks by a switching particle swarm optimization algorithm. Expert Syst. Appl..

[46] Zeng N., Wang Z., Zhang H., Alsaadi F.E. (2016). A novel switching delayed pso algorithm for estimating unknown parameters of lateral flow immunoassay. Cogn. Comput..

[47] Song Q., Wang Z. (2008). Neural networks with discrete and distributed time-varying delays: A general stability analysis. Chaos Soliton. Fract..

[48] Liu W., Wang Z., Liu X., Zeng N., Bell D. (2019). A novel particle swarm optimization approach for patient clustering from emergency departments. IEEE Trans. Evol. Comput..

[49] Zhan Z., Zhang J., Li Y., Chung H.S.H. (2009). Adaptive particle swarm optimization. IEEE Trans. Syst. Man Cybern. Part B-Cybern..

[50] Bergh F., Engelbrecht A.P. (2005). A study of particle swarm optimization particle trajectories. Inform. Sci..

[51] Liu X., Zhan Z., Gao Y., Zhang J., Kwong S., Zhang J. (2019). Coevolutionary particle swarm optimization with bottleneck objective learning strategy for many-objective optimization. IEEE Trans. Evol. Comput..

[52] Yao X., Liu Y., Lin G. (1999). Evolutionary programming made faster. IEEE Trans. Evol. Comput..

[53] Mirjalili S. (2015). The ant lion optimizer. Adv. Eng. Softw..

[54] Zhang L., Zhang L., Mou X., Zhang D. (2011). Fsim: A feature similarity index for image quality assessment. IEEE Trans. Image Process..

[55] Huynh-Thu Q., Ghanbari M. (2008). Scope of validity of PSNR in image/video quality assessment. Electron. Lett..

[56] He L., Huang S. (2017). Modified firefly algorithm based multilevel thresholding for color image segmentation. Neurocomputing.

[57] Aziz M.A.E., Ewees A.A., Hassanien A.E. (2017). Whale Optimization Algorithm and Moth-Flame Optimization for multilevel thresholding image segmentation. Expert Syst. Appl..

[58] Wang Z., Bovik A.C., Sheikh H.R., Simoncelli E.P. (2004). Image quality assessment: From error visibility to structural similarity. IEEE Trans. Image Process..

[59] Arbelaez P., Maire M., Fowlkes C., Malik J. (2010). Contour Detection and Hierarchical Image Segmentation. IEEE Trans. Pattern Anal. Mach. Intell..

[60] Bandyopadhyay R., Kundu R., Oliva D., Sarkar R. (2021). Segmentation of brain MRI using an altruistic Harris Hawks’ Optimization algorithm. Knowl.-Based Syst..

[61] Zeng N., Zhang H., Song B., Liu W., Li Y., Dobaie A.M. (2018). Facial expression recognition via learning deep sparse autoencoders. Neurocomputing.

[62] Gandomi A.H., Yang X., Alavi A.H. (2013). Cuckoo search algorithm: A metaheuristic approach to solve structural optimization problems. Eng. Comput..

[63] Faramarzi A., Heidarinejad M., Mirjalili S., Gandomi A.H. (2020). Marine Predators Algorithm: A nature-inspired metaheuristic. Expert Syst. Appl..

